# Expression of Cellulosome Components and Type IV Pili within the Extracellular Proteome of *Ruminococcus flavefaciens* 007

**DOI:** 10.1371/journal.pone.0065333

**Published:** 2013-06-04

**Authors:** Maša Vodovnik, Sylvia H. Duncan, Martin D. Reid, Louise Cantlay, Keith Turner, Julian Parkhill, Raphael Lamed, Carl J. Yeoman, Margret E. Berg. Miller, Bryan A. White, Edward A. Bayer, Romana Marinšek-Logar, Harry J. Flint

**Affiliations:** 1 Chair for Microbiology and Microbial Biotechnology, Biotechnical Faculty, University of Ljubljana, Ljubljana, Slovenia; 2 Rowett Institute of Nutrition and Health, University of Aberdeen, Aberdeen, United Kingdom; 3 Wellcome Trust Sanger Institute, Cambridge, United Kingdom; 4 Department of Biological Chemistry, The Weizmann Institute of Science, Rehovot, Israel; 5 Department of Molecular Microbiology and Biotechnology, Tel Aviv University, Ramat Aviv, Israel; 6 Department of Animal Sciences, University of Illinois at Urbana-Champaign, Urbana, Illinois, United States of America; 7 Institute for Genomic Biology, University of Illinois at Urbana-Champaign, Urbana, Illinois, United States of America; University Paris South, France

## Abstract

**Background:**

*Ruminococcus flavefaciens* is an important fibre-degrading bacterium found in the mammalian gut. Cellulolytic strains from the bovine rumen have been shown to produce complex cellulosome structures that are associated with the cell surface. *R. flavefaciens* 007 is a highly cellulolytic strain whose ability to degrade dewaxed cotton, but not Avicel cellulose, was lost following initial isolation in the variant 007S. The ability was recovered after serial subculture to give the cotton-degrading strain 007C. This has allowed us to investigate the factors required for degradation of this particularly recalcitrant form of cellulose.

**Methodology/Principal Findings:**

The major proteins associated with the bacterial cell surface and with the culture supernatant were analyzed for *R. flavefaciens* 007S and 007C grown with cellobiose, xylan or Avicel cellulose as energy sources. Identification of the proteins was enabled by a draft genome sequence obtained for 007C. Among supernatant proteins a cellulosomal GH48 hydrolase, a rubrerthyrin-like protein and a protein with type IV pili N-terminal domain were the most strongly up-regulated in 007C cultures grown on Avicel compared with cellobiose. Strain 007S also showed substrate-related changes, but supernatant expression of the Pil protein and rubrerythrin in particular were markedly lower in 007S than in 007C during growth on Avicel.

**Conclusions/Significance:**

This study provides new information on the extracellular proteome of *R. flavefaciens* and its regulation in response to different growth substrates. Furthermore it suggests that the cotton cellulose non-degrading strain (007S) has altered regulation of multiple proteins that may be required for breakdown of cotton cellulose. One of these, the type IV pilus was previously shown to play a role in adhesion to cellulose in *R. albus*, and a related pilin protein was identified here for the first time as a major extracellular protein in *R. flavefaciens.*

## Introduction

Bacteria related to *Ruminococcus flavefaciens* play an important role in the degradation of lignocellulosic plant fibre in the rumen and large intestine of herbivorous animals. Many *R. flavefaciens* strains isolated from the rumen are able to degrade insoluble crystalline cellulose present in test substrates such as filter paper and dewaxed cotton fibre. Studies over the past 10 years have demonstrated the presence of an elaborate cellulosome complex in this species [1], [2]. Thus all cellulolytic strains so far examined possess the *sca* gene cluster that encodes cell surface scaffoldin proteins shown to bind the enzymatic subunits of the cellulosome via specific cohesin-dockerin interactions [3], [4]. Information on the *R. flavefaciens* cellulosome has been greatly advanced by the genome sequence of strain FD1, which has revealed more than 200 putative cellulosome components [5], [6].

Many questions remain unanswered, however, not only concerning cellulosome organization but more generally about functional aspects of lignocellulose degradation by *R. flavefaciens*. One of the key differences between the cellulosome of *R. flavefaciens* and that of other cellulolytic species is the lack of an identified cellulose-binding module within the major cellulosomal scaffolding proteins of *R. flavefaciens* [4]. Instead, binding to cellulose has been assumed to be mediated via modules present in individual enzymes [7] and by the surface-attached protein CttA [8], but critical evidence on substrate binding by whole cells, as opposed to recombinant protein modules, has been lacking. Earlier work reported a mutational variant of *R. flavefaciens* strain 007C (referred to as 007S) that had lost the ability to degrade dewaxed cotton cellulose, while largely retaining the ability to degrade other forms of insoluble cellulose such as Avicel [9], [10]. This difference was thought likely to reflect differences in substrate adhesion resulting from changes in extracellular protein expression that potentially identify the key proteins involved [8]. Cellulosomal components are known to be subject to regulation in *R. flavefaciens*, since growth on cellulose compared to cellobiose leads to increased transcription of several cellulosomal scaffolding proteins and enzymatic subunits in strain FD1 [5]. Information on the abundance of individual proteins and on substrate-driven changes in extracellular protein expression has however been somewhat limited [8], [11].

This investigation examines the extracellular proteomes of *R. flavefaciens* strains 007C and 007S grown with crystalline cellulose (Avicel), xylan or cellobiose as energy sources. Our results reveal the up-regulation of several major cellulosome-associated proteins during growth on cellulose, consistent with evidence from the previous transcriptional studies in *R. flavefaciens* strain FD1 [5]. In addition however we detect major increases in a pilus-associated protein and in rubreryrthin in response to cellulose, and multiple differences in protein expression between the strain 007C and 007S that suggest new requirements for effective cellulose degradation in this species, including the likely involvement of type IV pili.

## Results

### Major Extracellular Proteins of *R. flavefaciens* 007C and 007S

*Ruminococcus flavefaciens* 007 was first isolated from rumen fluid as an actively cellulolytic culture able to degrade dewaxed cotton cellulose. This activity was lost upon subculture with cellobiose as the energy source, resulting in cotton-degrading (007C) and non-degrading (007S) derivatives [9], [10]. In the present study we investigated the major extracellular proteins produced by each of these strains during growth on alternative substrates. *Ruminococcus flavefaciens* 007C cultures were grown anaerobically on modified Hungate-Stack medium containing 0.4% cellobiose, 0.4% oat spelt xylan, 1% insoluble cellulose (Avicel PH101) or 0.1% dewaxed cotton as added energy sources. Strain 007S cannot grow on de-waxed cotton cellulose, but its growth with cellobiose, Avicel and oat spelt xylan is similar to that of 007C (Fig. S1, [9]). Cultures were harvested at two time points in stationary growth phase and processed as described in the Materials and Methods section to give concentrated cell culture supernatant (CCSUP), bacterial cell wall-associated (CWAP) and cellulose-bound (CBP) cell fractions. Fractions from two independent experiments were analyzed for each strain.

**Figure 1:**
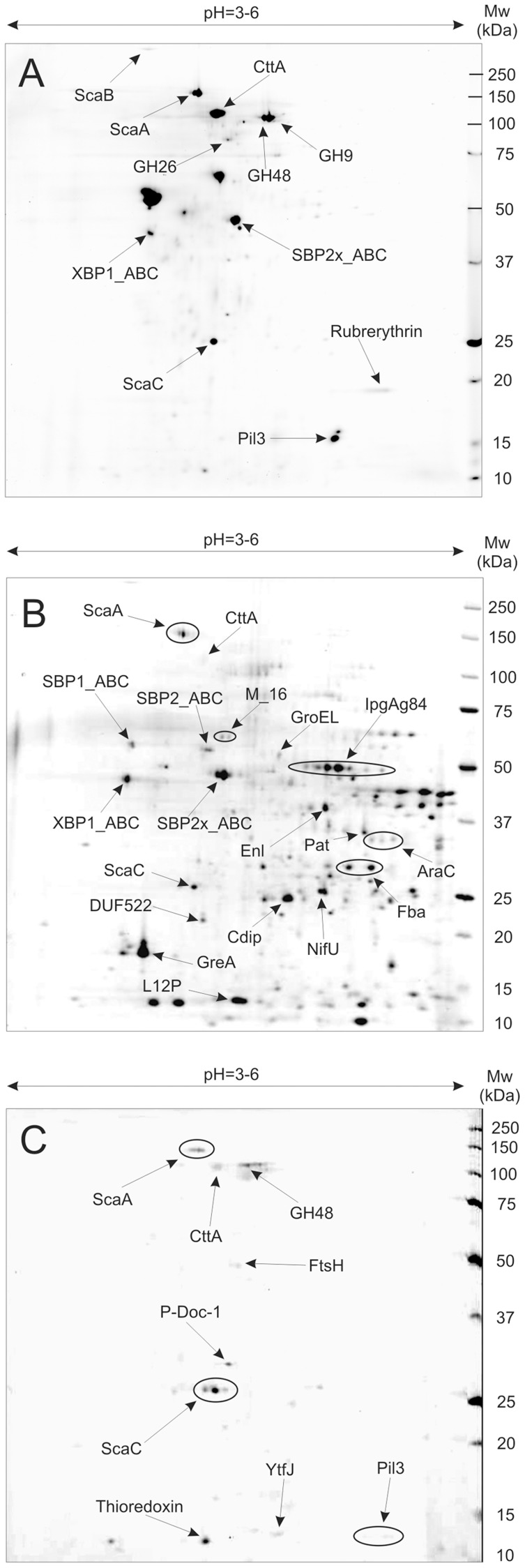
Proteome of cellulose-grown *Ruminococcus flavefaciens* 007C. Showing 2D gel separations of: A) cell culture supernatant (CCSUP) protein fraction, B) cell wall associated (CWAP) fraction (following growth on Avicel for 7.5 days); C) cellulose-bound (CBP) fraction following growth on dewaxed cotton for 9.5 days. Abbreviations: Pil3 - protein with type IV pilin N-terminal domain (encoded by *pil3*), P-Doc-1 - protein with dockerin type-1 (no other conserved domains/signature sequences have been detected), GH - glycoside hydrolase, SBP_ABC - substrate-binding component of ABC-type sugar transport system, XBP_ABC - xylose-binding component of ABC-type sugar transport system, SBP2x_ABC - substrate-binding component of ABC-type sugar transport system involved in xylan utilization, M_16– Zn-dependent peptidase, IpgAg84 - immunogenic protein antigen 84, GroEL - GroEL chaperonin, Enl – enolase, Pat - phosphate acetyltransferase, Fba - fructose-bisphosphate aldolase, NifU – NifU homolog involved in Fe-S cluster formation, GreA - transcription elongation factor GreA, Cdip - cell division initiation protein, DUF552 - putative conserved protein with unknown function (DUF552), AraC - transcriptional regulator, L12P - 50S ribosomal protein L12P, YtfJ - sporulation protein YtfJ, FtsH - ATP-dependent metalloprotease.

Major spots of the three fractions were excised after 2D gel separation of proteins from cellulose-grown cultures (Fig. 1) and identified by mass spectrometry (ESI-MS/MS); identities are listed in Tables S1–S5. Four of the five proteins encoded by the *sca* gene cluster (ScaA, ScaB, ScaC and CttA) were among the most prominent proteins detected in the culture supernatant from Avicel-grown cultures. A draft genome sequence obtained for *R. flavefaciens* 007C showed that the *sca* genes are arranged in the same order as in strains FD-1 and 17 [3]. The predicted *sca* gene products show close to 100% sequence identity with those of *R. flavefaciens* 17 (Table 1).

**Table 1:** *Sca* gene cluster of *R. flavefaciens* 007C and % amino acid identity (similarity) of encoded scaffoldins to the closest homologues from other strains.

ORF	Contig	ORF coordinates		Annotation	Protein homologues in other *R. flavefaciens* strains (in parentheses)	E-value	Coverage	Identity (similarity)
***capA***	00019	6163	7452	Putative capsule biosynthesis protein	CAL07961.1 (17)	0.0	98%	99.5%(100%)
					ZP_06144570.1 (FD-1)	2e-169	98%	69.0%(89.3%)
**scaC**	00019	4779	5627	ScaC cellulosomal scaffoldin protein precursor	CAE51046.2 (17)	3e-157	100%	100% (100%)
					CAQ16964.1 (JM1)	3e-116	100%	75.5% (90.5%)
					CAO00728.1 (C94)	5e-102	100%	65.4% (87.9%)
					ZP_06144572.1 (FD-1)	4e-86	96%	52.0% (77.2%)
**scaA**	00019	2039	4681	ScaA cellulosomal scaffoldin protein precursor	CAC34384.3 (17)	0.0	100%	98.6% (99.2%)
					CAO00729.1 (C94)	2e-147	99%	45.8% (72.1%)
					ZP_06144573.1 (FD-1)	1e-74	99%	30.7% (58.3%)
***scaB***	00019 00018	1 115883	2007 116710	ScaB cellulosomal scaffoldin precursor	CAC34385.1 (17)	0.0	99%	98.9% (99.1%)
					CAO00730.1 (C94 )	0.0	98%	57.2% (82.4%)
					ZP_06144574.1 (FD-1)	2e-102	98%	43.4% (74.7%)
***cttA***	00018	113280	115496	CttA carbohydrate-binding protein precursor	CAH18995.2 (17)	0.0	100%	98.3% (99.1%)
					CAO00731.1 (C94 )	0.0	98%	51.8% (80.2%)
					ZP_06144575.1 (FD-1)	2e-177	98%	44.2% (71.9%)
**scaE**	00018	11227	112976	ScaE cell-surface anchored scaffoldin protein precursor	CAH18996.1 (17)	1e-87	100%	98.7% (99.1%)
					CAO00732.1 (C94 )	7e-43	100%	61.2% (82.4%)
					ZP_06144576.1 (FD-1)	1e-27	100%	49.4% (70.3%)
***orfA***	00018	111468	112214	ATPase, PP family	CAL07962.2 (17)	1e-142	100%	98.7%(100%)
					Q14SX3_RUMFL (FD-1)	1e-99	100%	98.3%(100%)

**Figure 2:**
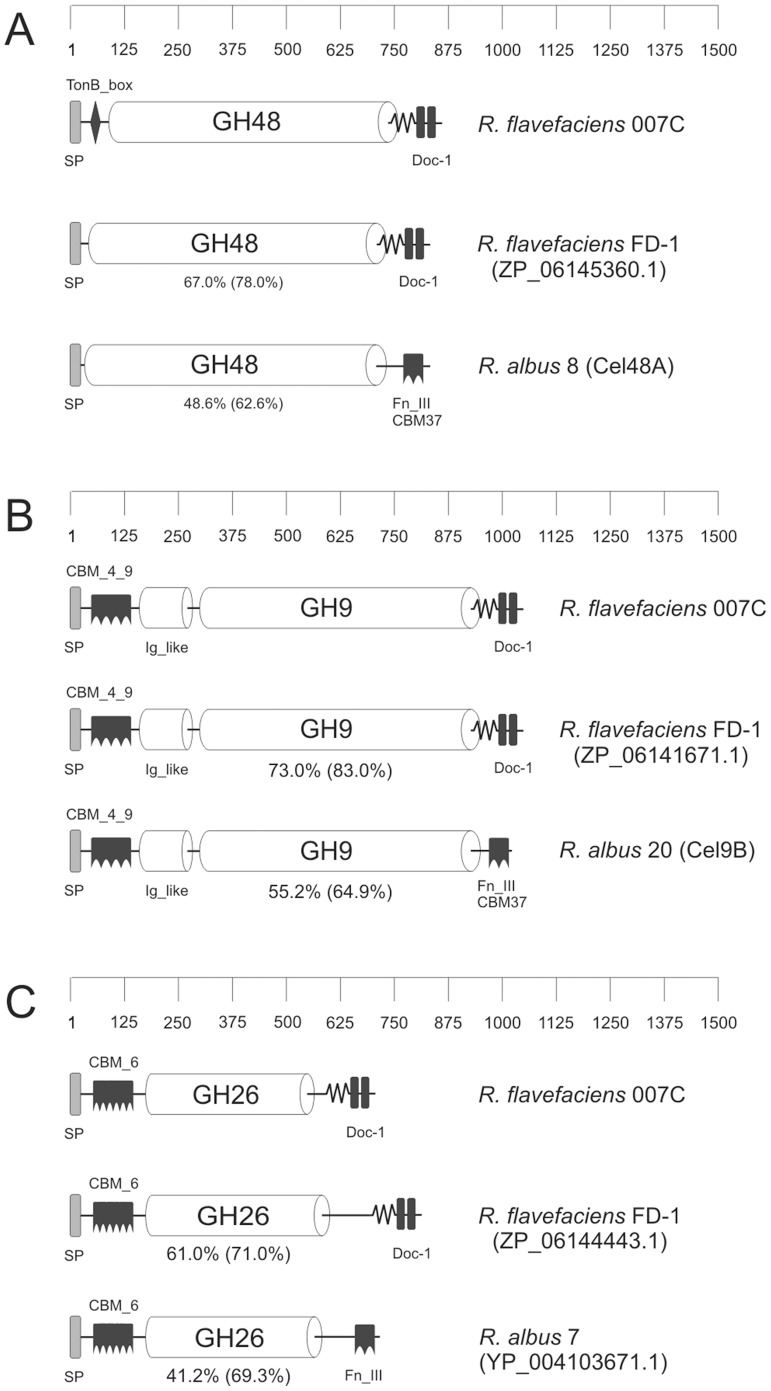
Modular structures of prominent glycoside hydrolases. These are compared with the predicted enzymes showing the highest % similarity (Blast) from *R. flavefaciens* FD-1 or *R. albus*: (A) glycoside hydrolase family 48; (B) glycoside hydrolase family 9; (C) glycoside hydrolase family 26. Abbreviations: SP - signal peptide, Doc-1 - dockerin type I, GH - glycoside hydrolase, CBM – carbohydrate-binding module, TonB_box: TonB-dependent receptor protein signature 1, Fn_III – fibronectin type III domain, Ig-like – immunoglobulin-like domain. The numbers below the *R. flavefaciens* FD-1 and *R. albus* enzymes represent % amino acid identity to the *R. flavefaciens* 007C homologue.

In addition enzymes carrying GH48, GH9 and GH26 catalytic domains as well as type 1 dockerin domains (Doc-1) were identified among the major proteins in culture supernatants. These proteins show a close resemblance to homologues from *R. flavefaciens* strain FD-1. The catalytic domains also showed a high degree of amino acid sequence identity (>40%) with major extracellular enzymes from *R. albus* that lack dockerins (Fig. 2). Additional proteins identified in the three fractions are listed in Tables S1–S5, and several of these are discussed further below.

### Influence of Growth Substrate on Expression of Major Extracellular Proteins

**Figure 3:**
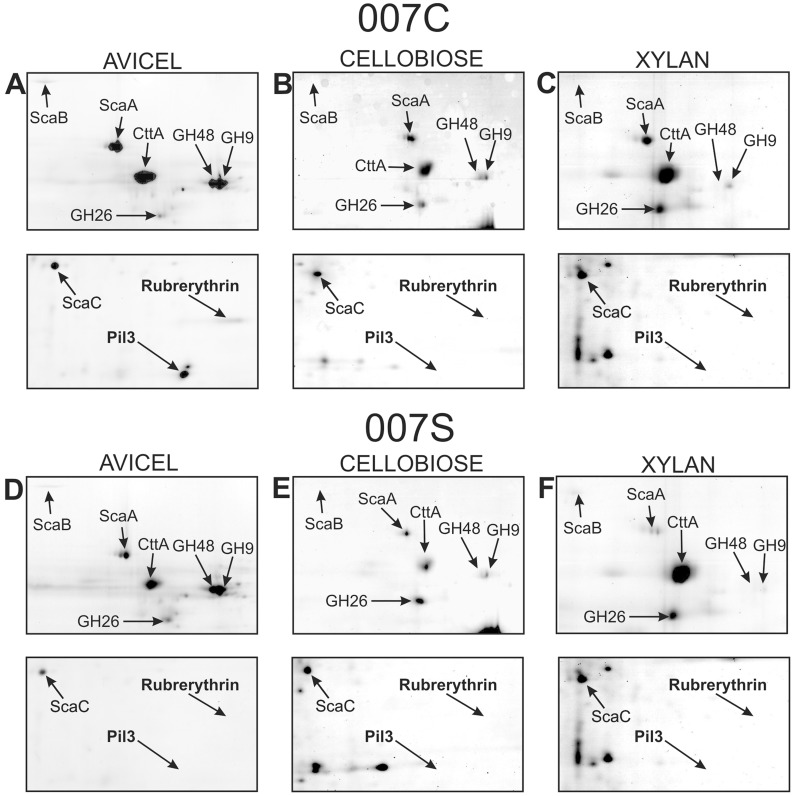
Impact of growth substrate on the extracellular proteome. Expanded views are show for 2D gel separations of supernatant proteins from *R. flavefaciens* 007C and 007S cutures grown on Avicel (7.5 days), xylan (48 hours) or cellobiose (24 hours). The arrows point to identified proteins; relative expression levels for some of these are shown in Figure 4.

We detected strong up-regulation of three proteins in culture supernatants of strain 007C (>10 fold) that was specific to Avicel-grown relative to cellobiose-grown cultures, with little or no increase in xylan-grown cultures (Fig. 3, 4A). These proteins were a GH48 cellulase, a type IV pilin homologue and a rubrerythrin-like protein; in contrast, the cellulosomal proteins ScaC (Fig. 3) and GH9 and GH26 enzymes (Fig. 3, 4A) were less affected by the growth substrate. There was some evidence for substrate-dependent expression of CttA and ScaA, especially among cell wall associated proteins (Fig. S2), but these appeared to increase in both xylan and Avicel-grown cultures relative to cellobiose.

**Figure 4:**
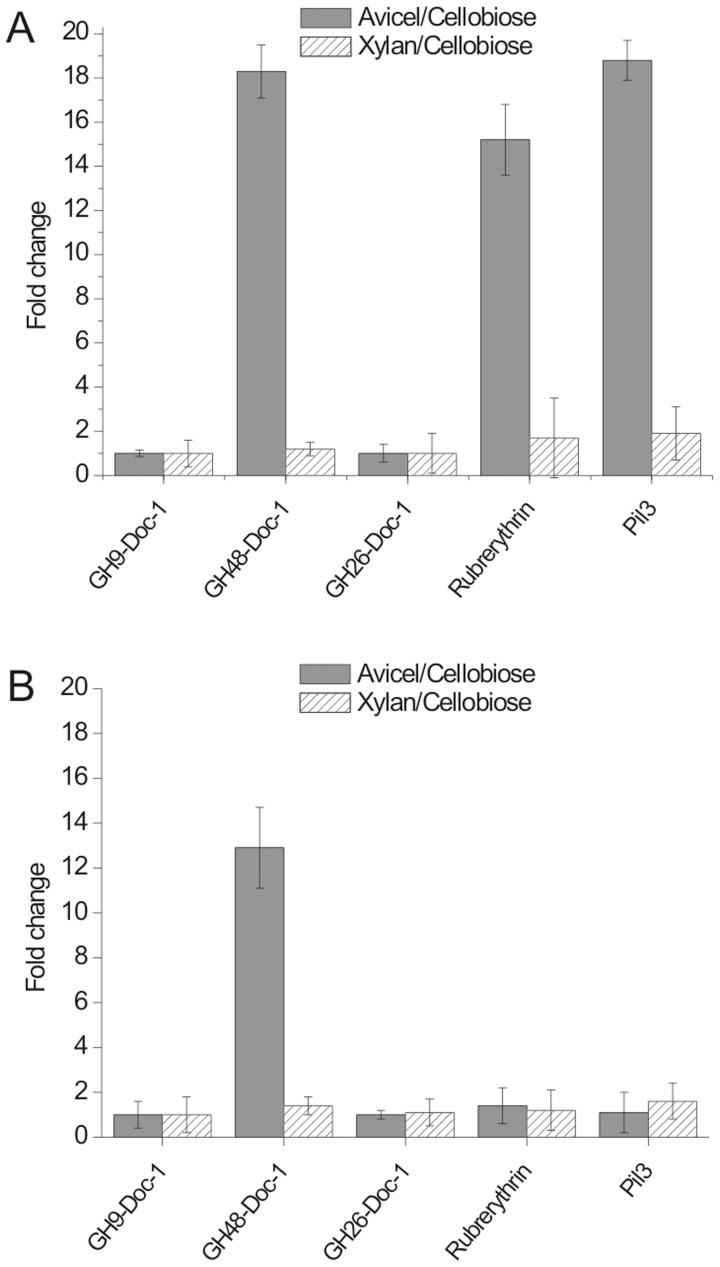
Cellulose-specific changes in expression of culture supernatant proteins in *R. flavefaciens* strains grown on Avicel (7.5 days), xylan (48 hours) or cellobiose (24 hours). (A) supernatant (CCSUP) fraction of strain 007C, (B) supernatant fraction of strain 007S. 1-fold change indicates no change, >1-fold change indicates upregulation in comparison to cellobiose-grown cultures, <1-fold change indicates downregulation in comparison to cellobiose-grown cultures. Results are based on duplicated biological experiments, and three technical replicates for each gel separation.

### Comparison of Extracellular Protein Expression between 007C and 007S

Protein expression was compared for the two strains 007C and 007S after the same period of growth on each substrate, based on the mean of two independent experiments. The strains did not differ significantly for the cell wall-associated protein fraction (Fig. S2). Differences were however detected for the culture supernatant fraction (Fig. 3, Fig. 4), including two of the proteins that showed the largest degrees of induction by Avicel in strain 007C, the type IV pilin and rubrerythrin homologues. Interestingly the type IV pilin protein was also detected in the cotton-bound fraction of *R. flavefaciens* 007C (Fig. 1C, Table S4).

### Type IV pilin Gene Cluster in *R. flavefaciens* 007

The 14-kDal protein identified in *R. flavefaciens* supernatant fluids shows N-terminal homologies with type IV pilins of several Gram-negative bacteria *(Moraxella bovis*, *Pseudomonas aeruginosa*, *Dichelobacter nodosus*, *Neisseria meningitidis*, *Myxococcus xanthus*, *Eikenella corrodens*, *Neisseria gonorrhoeae* in *Aeromonas hydrophila, Deinococcus radiodurans, Vibrio vulnificus, Photobacterium profundum*) (Fig. S3). This protein also showed 55.6% amino acid sequence identity with pilA2 from *Ruminococcus albus* 20. From the location of prepilin peptidase recognition motif (ĜFxxxE) detected in the sequence of *R. flavefaciens* 007 pilin, it is possible to infer that the protein is synthesised with a 7-residue leader sequence (MKTTKKG), that is cleaved to release a 165-residue mature polypeptide with a theoretical pI of 5.75 and MW of 13435 Da. These values do not correspond exactly with the position observed on the 2D gel of supernatant protein fraction, probably indicative of post-translational modification (PTM), as reported for the *R. albus* pilin [12], [13]. The shift toward higher MW that is accompanied by lowering of protein pI is often associated with the addition of phosphate groups, which was supported by NetPhosK analysis of Pil3 sequence revealing 17 possible phosphorylation sites. Interestingly, the properties of the same protein in CBP fraction appear to be somewhat closer to the calculated values, indicating lower level (or absence) of PTMs. This may affect protein polymerization and formation of visible structures, as in the case of *N. meningitidis*. In these bacteria, the major subunit of type IV pili is post-translationally modified by the addition of phosphoglycerol at Ser93, which results in increasing its negative charge leading to destabilization of the pili and detachment of bacteria from the microcolonies on host tissue in certain periods of growth cycle [14].

**Figure 5:**
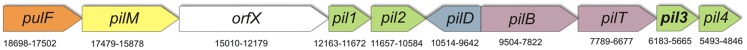
Type IV pili biogenesis cluster *of R. flavefaciens* 007C. The protein Pil3, encoded by the ***pil3*** gene, shares 51% amino acid identity to the cellulose-binding protein (CbpC) of *R. albus* 8 and 43% amino acid identity to *R. albus* 20 protein GP25 (a protein that was shown to be underproduced by spontaneous cellulose adhesion-defective mutant D5). The gene downstream (*pil4*) encodes another protein with prepilin-type IV N-terminal domain, whereas the genes upstream encode: putative PilT−/PilB-like ATPases (*pilT, pilB*), prepilin peptidase (*pilD*), two proteins with pseudopilin PulG-like domains (*pil1*, *pil2*), type IV pilus assembly protein (*pilM*) and a type II secretion system protein (*pilF*). OrfX refers to open reading frame without recognized signal sequences.

The *R. flavefaciens pil3* gene is flanked by open reading frames that are homologous to genes known to be involved in the assembly and secretion of type IV pili and flagella (Fig. 5, Table S6). These include upstream genes encoding a putative pilus retraction (PilT-like) ATPase, followed by a PilB (Vir B11)-like ATPase, and a putative prepilin peptidase (PilD-like) gene. The first two genes in the cluster encode a putative PilM-like pilus assembly protein (involved in the export of the pilus subunit and its assembly in *P. aeruginosa*) and a putative F-domain of the type II secretion system (PulF homologue). One gene (*pil4*) with a type IV pilus signature sequence (prepilin-type N-terminal cleavage/methylation site) is present downstream of *pil3*, and also encodes a protein with high similarity to *R. albus* pilin CbpC. The nucleotide composition of the putative pilus biogenesis cluster did not differ significantly in its G+C content (47.04%) from the whole genome (45.2%). We also detected a homologous gene cluster in the genome of *R. flavefaciens* FD1 [5].

**Figure 6:**
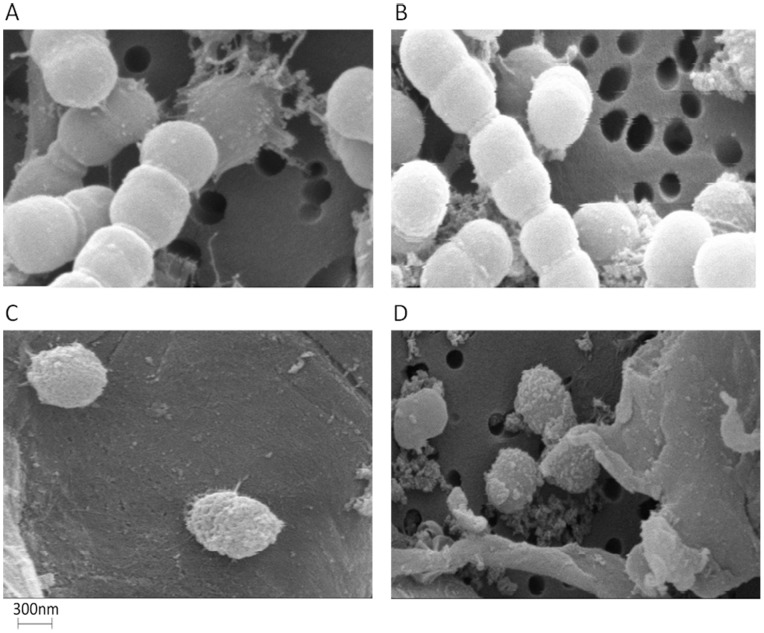
*R. flavefaciens* 007 cell morphology revealed by scanning electron microscopy. Cells grown on cellobiose: (A) 007C, (B) 007S; or on cellulose: (C) 007C, D) 007S.

Figure 6 shows the morphology of cells of 007C and 007S grown on cellobiose or cellulose as revealed by scanning EM. Contact between the bacterial extracellular matrix and the cellulose surface is evident particularly for strain 007C.

## Discussion

The *sca* gene cluster of *R. flavefaciens* 007C is almost identical to that reported in *R. flavefaciens* 17 [4], suggesting that this strain will show a similar cellulosome organization. The proteomic analysis conducted here reveals that four *sca*-encoded proteins, ScaA, ScaB, CttA and ScaC, are among the major supernatant and cell-associated proteins detected in Avicel-grown cultures. The fifth *sca* protein (ScaE) is covalently bound to the bacterial cell wall in the closely related *R. flavefaciens* strain 17 and would therefore not be expected to appear in the fractions examined here [11]. In addition, three dockerin-carrying enzymes, a GH48, GH9 and GH26, were prominent in culture supernatant fluids of Avicel-grown cells. Up-regulation of the major exo-acting GH48 has also been observed in *C. thermocellum* during growth on cellulose [15]–[18] and this enzyme is known to play a key role in cellulose breakdown. A prominent GH44 enzyme present in the proteome of xylan-grown *R. flavefaciens* 17 [19], although encoded by the genome, was not detected here in *R. flavefaciens* 007.

Regulation of *sca* cluster gene expression at the mRNA level has been examined in *R. flavefaciens* FD-1 by Berg Miller *et al.* [5] who reported approximately 4.5 fold up-regulation of *scaA*, *scaB* and *scaC* mRNA in cells grown on cellulose in comparison to cellobiose. Our study suggests up-regulation of ScaA and ScaB proteins in *R. flavefaciens* 007C, although ScaC was unaffected by the growth substrate. The previous study [5] also detected considerable up-regulation of several multimodular xylanases, but did not report upregulation of the GH48 enzyme that was seen here. The differences between the two studies might perhaps reflect inter-strain differences. On the other hand the previous study examined mRNA expression in cells at early growth stages with a different type of cellulose substrate (9 h of growth on cellobiose and 19 h growth on filter paper) while we have reported protein levels in different cell fractions in stationary cultures (after 14 h growth on cellobiose and 180 h growth on Avicel). Differences in temporal gene expression, protein secretion and protein turnover could therefore all be involved in explaining these differences. Differential regulation in response to the growth substrate has also been reported recently for *C. cellulovorans* cellulosomal proteins [20].

An important new finding from this study is the detection of an abundant type IV pilin protein both in Avicel-grown culture supernatants and among proteins attached to cotton cellulose in *R. flavefaciens* 007C. Furthermore this protein was specifically upregulated (19 fold) by growth of *R. flavefaciens* 007C on Avicel compared with cellobiose, suggesting that it has a role in cellulose breakdown. Type IV pili have also been shown to be expressed in the related cellulolytic species *R. albus* where they are reported to have a role in binding bacterial cells to cellulose [13], [21]–[23]. Interestingly the evidence for this was based on studies on variants of two different strains of *R. albus* that had been selected for loss of adhesion to cellulose [13], [23]. Similarly, a cellulose binding protein identified as type IV pilin was found to be missing in cellulose adhesion-defective mutants of *Fibrobacter succinogenes* [24]. Pili composed of type IVa subunits are found in a broad range of Gram-negative bacteria with disparate host and tissue specificity, but have also been reported from some Gram-positive species [25]. They apparently play different physiological roles: twitching motility, host cell adhesion and immune escape, microcolony formation and DNA uptake.

Another novel finding was the strong up-regulation of a protein annotated as a putative rubrerythrin-like protein during growth on Avicel. This 20-kDa protein has a conserved rubredoxin-type Fe(Cys)_4_ site at the N-terminal region and a ferritin-like di-iron domain at the C-terminus. Rubrerythrin is a non-heme iron enzyme, widely distributed in anaerobic bacteria and archaea [26]–[28]. Although it would normally be assumed to be intracellular, it accumulated in the supernatant fluids of *R. flavefaciens* 007C cultures and was strongly upregulated by growth on cellulose. Various activities of rubrerythrin-like proteins have been described in bacteria [29], including pyrophosphatase, ferroxidase and superoxide dismutase activities [26]. However, recent reports suggest that its true function may be as a H_2_O_2_ reductase as part of an alternative detoxification system in strict anaerobic bacteria and archaea [30]. Recently, expression of a small rubrerythrin-like protein in *Clostridium acetobutylicum*, Hsp21, was shown to be induced not only by oxidative stress, but also by various other stress factors, such as salt, increased pH, high concentration of solvents or cold shock [31]. The possible significance of this rubrerythrin-like protein for growth of *R. flavefaciens* on cellulose is an intriguing question for future research.

The strain 007S differs from 007C in its cell morphology as well as in its inability to grow on cotton cellulose, while retaining the ability to degrade Avicel [9], [10]. Strain 007S has been shown here to display several quantitative protein expression changes by comparison with 007C. In particular, two proteins, the type IV pilus and the rubrerythrin-like protein showed markedly lower up-regulation in Avicel-grown relative to cellobiose-grown cultures of 007S compared with 007C. It seems probable that the loss of cotton cellulose degradation observed in 007S results from a regulatory mutation that exerts pleiotropic effects that include reduced expression of key adhesion factors. A previous report indicated reduced expression of the substrate-binding protein CttA found in cellulose-attached proteins from 007S compared with that of strain 007C [11]. The present study however revealed little difference between 007S and 007C in CttA expression among total supernatant proteins.

The fact that the cotton non-degrading variant 007S showed reduced expression of the *pil3* gene suggests that type IV pili, although yet to be demonstrated directly in this species, might play a special role in the degradation of highly crystalline cellulose by *R. flavefaciens*. Adhesion of *R. flavefaciens* to insoluble cellulosic substrates appears to involve multiple adhesion factors including type IV pili, specialist adhesion proteins (CttA) [7] and CBMs present in individual enzymes [8]. Multiple adhesion mechanisms have also been proposed in *R. albus* [32] although there has been no report of a CttA-like protein in that species. The relative importance of these different binding mechanisms is likely to vary with the nature of the substrate and the proximity of cells and enzyme complexes to the surface of the substrate. Since the 007S variant is still able to degrade Avicel, this suggests that type IV pili could be particularly important in adhesion to highly crystalline celluloses such as cotton cellulose.

In conclusion this study has used proteomic analysis to examine the expression of major extracellular proteins relevant to lignocellulose breakdown in *R. flavefaciens* 007. Our findings identify the major cellulosome-associated structural and catalytic proteins within the extracellular proteome, but also reveal for the first time a likely role for type IV pili in cellulolysis by *R. flavefaciens*. These appendages are proposed to play a role in the adhesion to cellulose by *R. albus*, wherein there has been no direct evidence for cellulosome organization, and the type IV pili are now candidates for contributing to cellulose binding also in the cellulosomal species, *R. flavefaciens*.

## Materials and Methods

### Strains and Growth Conditions

*R. flavefaciens* 007C and 007S were grown anaerobically in 800 ml of Hungate-Stack medium [33] modified by the addition of 5% clarified rumen fluid. Medium was supplemented with 1% (wt/vol) Avicel PH101 (Honeywill & Stein, London, UK), 0.4% oat spelt xylan, 0.4% cellobiose (Sigma-Aldrich) or 0.1% (wt/vol) dewaxed cotton as energy sources. The cells were grown statically at 37°C until the beginning of stationary phase. Extracellular proteins were harvested at two or three time points of incubation on each substrate: after 14 and 24 hours of growth on cellobiose, 26 and 48 hours of growth on oatspelt xylan, 7.5 and 9.5 days of growth on Avicel and after 9.5, 11.5 and 13.5 days of growth on dewaxed cotton, which corresponded to the periods when highest specific avicelase activity and total protein concentration was measured during growth of the cultures (procedure below, Fig. S1).

### Total Protein and Avicelase Activity

To estimate the growth and cellulase activity on different substrates, the *R. flavefaciens* strains 007C and 007S were grown in duplicates of 8 ml modified Hungate-Stack supplemented with carbon sources as described above. After chosen periods of growth (between 10–48 hours for cultures grown on cellobiose, 10–78 hours for cultures grown on xylan and 1–15 days for cultures grown on cellulose substrates) the tubes were centrifuged at 9000 g, at 4°C, and the supernatants were separated from the pellets and frozen following the addition of 80 µL of protease inhibitors cocktail (PIC; Sigma Chem. Co., St. Louis MO). The pellets were washed with 2 ml of 50 mM Na-phosphate buffer pH 6.5 (NaPB), added 50 µl of PIC and resuspended in 1 ml of sterile MilliQ. To determine the rate of Avicel degradation by different fractions, 40 µl aliquot of each supernatant or pellet each sample was mixed with 230 µl of 1% (w/v) Avicel suspension in NaPB containing 1 mM CaCl_2_ and incubated 120 min at 37°C. After incubation, the reaction was stopped by 30 µl of 10% TCA and the mixture was centrifuged at 10,000 g. The concentration of reducing sugars in the supernatant was measured by the Lever method [34] using glucose as a standard. Total protein concentrations were determined by the method of Bradford using the Coomasie Plus kit (Pierce, Thermo Scientific).

### Extracellular Protein Fractionation

Cell culture supernatant fluids were removed after centrifuging the cultures at 10,000 g (4°C for 15 min), then dialyzed (four times against 4 L of distilled water, at 4°C), freeze dried and resuspended in 10 ml of distilled water (dH_2_O). Cell culture supernatant protein fraction (CCSUP) was then stored at −20°C pending analysis. Cell wall-associated protein (CWAP) and cellulose-bound protein (CBP) enriched fractions were prepared by slight modifications of previously published procedures [11], [35]. When preparing the cultures grown on cellulose, the cells were first separated from the substrate by differential settling. Following vigorous shaking, the substrate was left to sediment for 10 min. The cell culture supernatant fluid (containing detached cells) was then removed using a vacuum pump and centrifuged at 10,000 g, 4°C for 15 min. Cell pellets were washed twice with 100 ml TBS (25 mM Tris-HCl, 150 mM NaCl, pH 7.0) and centrifuged at 10,000 g (4°C, 15 min). The pellet from the final centrifugation step was resuspended in 40 ml of 4.5 mM MgSO_4_ in TBS with the addition of 2 µg DNAse I and RNAse A ml^−1^ and incubated at room temperature for 45 min. The pellet from another centrifugation (10,000 g, 4°C, 15 min) was resuspended in 14 ml of sarcosyl buffer (2% N-lauryl sarcosine, 1 mM protease inhibitor cocktail (Sigma Chem. Co., St. Louis MO), 1 mM PMSF (Sigma) in TBS) and incubated on ice for 1 h (with 3 short vortexing steps). After centrifugation at 15,000 g (4°C, 15 min) the supernatant was recovered and ultracentrifuged at 75,000 g, 4°C for 1 h (Optima Max ultracentrifuge, MSL-50 rotor, Beckman-Coulter, Fullerton, CA). This final supernatant fraction was 100× concentrated by ultrafiltration (Amicon-15, Millipore Inc., Billerica). The retentate (CWAP) was stored at −20°C pending analysis.

To obtain the CBP fraction residual Avicel/cotton was vigorously washed five times with 40 ml of TBS-Ca-Tween buffer (25 mM Tris-HCl, 150 mM NaCl, 1 mM CaCl_2_; pH 7.0). CBPs were recovered by incubating washed cotton/Avicel with 2% (w/vol) CHAPS at 70°C for 1 h, followed by heating at 100°C for 5 min. Supernatant fluids were 100× concentrated by ultrafiltration (Amicon-15) and stored at −20°C pending analysis.

### Two Dimensional Gel Electrophoresis and Image Analysis

All results relating to protein expression presented in this paper are based on duplicated biological experiments, with three technical replicates for each gel separation. Protein concentrations of CCSUP and CWAP fractions were measured by the BCA assay (Pierce Chem. Co., Rockford, IL). An aliquot of each sample containing 350 µg of protein was mixed with isoelectric focusing buffer (9 M urea, 4% CHAPS, 0.5% Biolite Ampholite; pH 3–10) and used for rehydration of 17 cm IPG strips, pH 3–6. Rehydration was performed on a Bio-Rad IEF cell at 20°C for 1 h without applied voltage, then at 50 V/strip for another 16 h. Initial start-up and ramping was performed according to the BioRad instruction protocol. IPG strips were incubated in equilibration buffer I (6 M urea, 2% SDS, 0.375 M Tris-HCl, pH 8.8, 20% glycerol, 130 mM dithiothreitol) and then in equilibration buffer II (6 M urea, 2% SDS, 20% glycerol, 135 mM iodoacetamide, 0.375 M Tris-HCl; pH 8.8) for 15 min at room temperature. Gradient (8–16%) SDS PAGE gels were prepared and cast on an Anderson IsoDalt casting apparatus. The gels were run at 200 V for 9.5 h, then fixed and stained by Colloidal Coomasie Blue staining as previously described [19]. The gels were imaged with a Bio-Rad GS-800 Scanning Densitometer and then analysed with Progenesis Samespots software (Nonlinear Dynamics) with incorporated statistics package). Differences in protein expression were considered significant when the following criteria were met: 1) differences in average normalized spot volumes were >5-fold and Anova p-values <0.05; or 2) differences in average normalized spot volumes were >1.5 and p-values <0.025.

### Spot Cutting, Trypsinization, Protein Identification by NANO LC MS/MS

The spots of interest (average spot volumes >5 e^+006^) were excised from the gels manually, placed in a 96-well V-bottom plate and trypsinized by using MassPrep station. The samples were analyzed using a nano LC system (LC Packings, Camberly, Surrey, UK) consisting of an ‘Ultimate’ nano LC system, with a column flow rate of 0.3 µL min^−1^, a ‘Famos’ autosampler set to an injection volume of 10 µL and a ‘Switchos’ microcolumn switching device with a flow rate 0.03 mL/min. The solvent used by the ‘Switchos’ was 0.1% formic acid. The nanocolumn was a C18 PepMap 100, 15 cm×75 µm i.d., 3 µm, 100 Å (LC Packings). HPLC grade solvents were used, 2% acetonitrile and 0.1% formic acid (A) and 80% acetonitrile and 0.08% formic acid (B). The gradient started at 5% B, proceeding to 95% B over 32 min, maintained for 10 min, then restored to 5% B for an additional 18 min giving a total run time of 60 min. The system was equilibrated at 95% A for 3 min prior to injection of subsequent samples.

The mass spectrometry was performed using a Q-Trap (Applied Biosystems/MDS Sciex, Warrington, UK) triple quadrupole fitted with a nanospray ion source, where Q3 is operated as a linear ion trap (LIT). The nanospray needle voltage was set at 2800 V. Oxygen Free Nitrogen (OFN) was used as the curtain gas and the collision gas. In the survey scan mode, the mass range in Q1 was set to m/z 450–1450 with a scan rate of 4000 amu/s. The criteria for selection of ions for fragmentation (Q2) were ions of 1.5^4^ cps or above. The collision energy was compound dependent (set to a maximum of 80 eV). The trap fill time (Q3) was set to a maximum of 250 ms and the scan rate was 4000 amu/s.

### *R. flavefaciens* 007C Genome Sequence

The *R. flavefaciens* 007C genome was sequenced with 55× coverage and assembled in 39 contigs by the Wellcome Trust Sanger Institute. The sequence is publicly available and was downloaded from their web site: ftp://ftp.sanger.ac.uk/pub/pathogens/Ruminococcus/flavefaciens/007c/Rflav007c.091211.seq.

### Protein Identification and Data Analysis

The total ion current (TIC) data were submitted for NCBInr database searching using the MASCOT search engine (Matrix Science) with the following search criteria: allowance of 0 or 1 missed cleavages; peptide mass tolerance of ±1.5 Da; fragment mass tolerance of ±1.5 Da, trypsin as digestion enzyme; carbamidomethyl fixed modification of cysteine; methionine oxidation as a variable modification; and charged state as 2^+^ and 3^+^. Identified proteins from different *R. flavefaciens* strains (mainly FD-1 and N17) were used to query the *R. flavefaciens* 007C local database for ORFs encoding orthologues in this strain (http://www.ncbi.nlm.nih.gov/projects/gorf/). Protein samples with MASCOT scores lower than 100, were subjected to *'de novo'* peptide sequencing, followed by search for best matching open reading frames inside the *R. flavefaciens* 007 genome contigs arranged in the local database (tBlastn, BioEdit [36]). The translated open reading frames were searched for conserved domains/motifs and annotated using BlastP (www.ncbi.com), Conserved domains, HMMPfam, BlastProDom, HMMSMART, HMMPIR, Superfamily, SignalPHMM/NN, TMHMM, pattern Scan (Prosite), HMMPIR, HMMPanther and HMMSMART (http://www.ebi.ac.uk/Tools/InterProScan/). Signal peptides typical for Gram-positive bacteria were predicted using SignalP 4.0 software (http://www.cbs.dtu.dk/services/SignalP/). Theoretical MW and pIs of all identified *R. flavefaciens* 007C proteins (without signal sequences) were calculated using Compute pI/MW tool on the Expasy server. Protein sequence similarities were calculated using FASTA sequence alignment software (http://www.ebi.ac.uk/).

### Analysis of type IV Pilus Biogenesis Genes of *R. flavefaciens* 007C

Protein with high N-terminal amino acid similarity to type IV pilins of several bacteria was additionally analyzed using Domain Enhanced Lookup Time Accelerated BLAST (DELTA-BLAST) algorithm and aligned with similar type IV pilins from other bacterial species by Clustal Ω (www.clustal.org/omega/). The alignment was visualized by Jalview software (www.jalview.org/). The genomic environment of the gene encoding the putative pilin was queried by a combination of ORF Finder and BlastP programs to discover putative genes involved in pili biogenesis. Conserved domains and motifs of the translated products were determined as stated above. Phosphorylation sites in putative type IV pilin were predicted by NetPhosK 1.0 software [37].

### Preparation of *R. flavefaciens* Cells for Electron Microscopy

Cultures of *R. flavefaciens* 007c and 007s were incubated anaerobically as described above with either cellobiose (18 h) or Avicel cellulose (48 h). Cells were pelleted by centrifugation at 1 500 g for 10 min, fixed with glutaraldehyde and post fixed with osmium tetroxide as described previously [10]. The mounted samples were coated in a thin layer of gold and were observed using a Zeiss EVO MA10 scanning electron microscope.

## Supporting Information

Figure S1 **Total cell protein concentrations (c_prot_) and specific avicelase activities of cell (SAA Cel) and supernatant (SAA Sup) fractions during measured during growth of** ***R. flavefaciens*** **007C and 007S on cellobiose, xylan, Avicel and dewaxed cotton.**
(DOCX)

Figure S2 **Major extracellular proteins identified in the cell wall associated (CWAP) fraction of** ***R. flavefaciens*** **007C and 007S grown with cellobiose (24 h), xylan (48 h) or Avicel cellulose (7.5 d) as energy sources.** Proteins referred to in Figure 4 are indicated by arrows.
(TIF)

Figure S3 **Clustal Ω alignment of N-terminal part of Pil3 (putative type IV pilin of** ***R. flavefaciens*** **007C) with type IV pili from Moraxella bovis,** ***Pseudomonas aeruginosa, Dichelobacter nodosus, Neisseria meningitidis, Myxococcus xanthus, Eikenella corrodens, Neisseria gonorrhoeae*** **in** ***Aeromonas hydrophila.***
(DOCX)

Table S1 **Major proteins identified in the cell culture supernatant (CCSUP) fraction of** ***R. flavefaciens*** **007C grown on Avicel for 7.5 days.**
(PDF)

Table S2 **Major extracellular proteins identified in the cell wall associated (CWAP) fraction of** ***R. flavefaciens*** **007C grown on Avicel for 7.5 days.**
(PDF)

Table S3 **Major extracellular proteins identified in cellulose-bound (CBP) fraction of** ***R. flavefaciens*** **007C grown on Avicel for 7.5 days.**
(PDF)

Table S4 **Major extracellular proteins identified in cellulose–bound (CBP) fraction of** ***R. flavefaciens*** **007C grown on dewaxed cotton for 9.5 days.**
(PDF)

Table S5 **Major proteins identified in cell culture supernatant (CCSUP) fraction of** ***R. flavefaciens*** **007C grown on dewaxed cotton for 9.5 days.**
(PDF)

Table S6 **Type IV pili biogenesis cluster of** ***R. flavefaciens*** **007C and three closest homologues of putative gene products.** Abbreviations: SP - signal peptide, TMR(s) - transmembrane region(s).
(PDF)
