# Correction: Expression of Cellulosome Components and Type IV Pili within the Extracellular Proteome of *Ruminococcus flavefaciens* 007

**DOI:** 10.1371/annotation/fed83700-d3cd-428e-ae52-e60524c97529

**Published:** 2013-12-23

**Authors:** Maša Vodovnik, Sylvia H. Duncan, Martin D. Reid, Louise Cantlay, Keith Turner, Julian Parkhill, Raphael Lamed, Carl J. Yeoman, Margret E. Berg. Miller, Bryan A. White, Edward A. Bayer, Romana Marinšek-Logar, Harry J. Flint

This paper was republished on December 23 due to a figure being erroneously omitted from the PDF. Please see the updated PDF with all figures included. 

