# Supplementary material for: Expression of Cellulosome Components and Type IV Pili within the Extracellular Proteome of Ruminococcus flavefaciens 007
Source: PLoS One. 2013 Jun 4;8(6):e65333. doi: 10.1371/journal.pone.0065333 (PMC3672088; doi:10.1371/journal.pone.0065333)
Supplement: Figure S1 — Total cell protein concentrations (cprot) and specific avicelase activities of cell (SAA Cel) and supernatant (SAA Sup) fractions during measured during growth of R. flavefaciens 007C and 007S on cellobiose, xylan, Avicel and dewaxed cotton. (DOCX) [file pone.0065333.s001.docx]

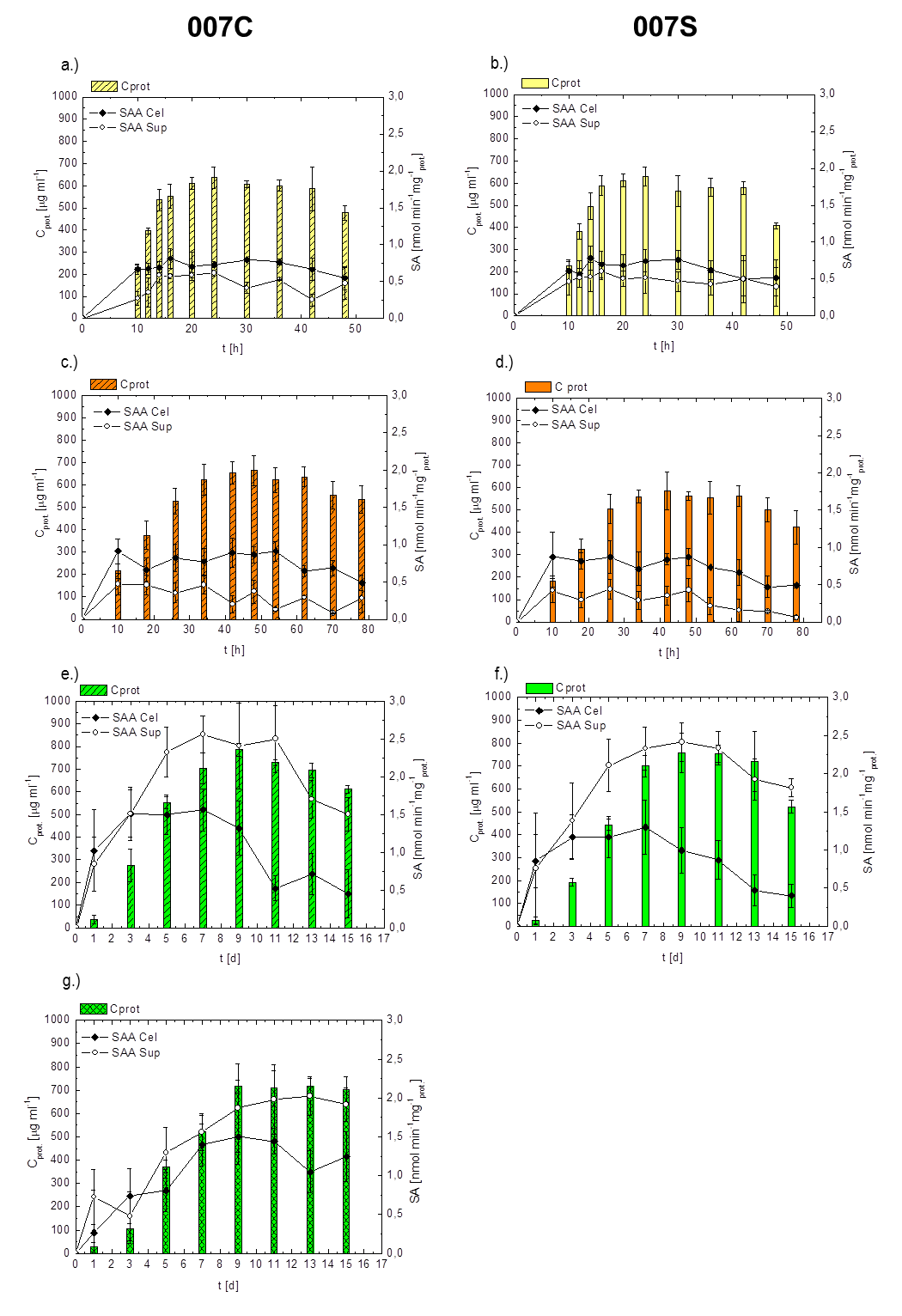
**Figure S1.** Total cell protein concentrations (c_prot_) and specific avicelase activities of cell (SAA Cel) and supernatant (SAA Sup) fractions during measured during growth of *R. falvefaciens* 007C and 007S on cellobiose (a., b.), xylan (c., d.), Avicel (e., f.) and dewaxed cotton (g.).
