# Supplementary figures and images for: Expression of Cellulosome Components and Type IV Pili within the Extracellular Proteome of Ruminococcus flavefaciens 007
Source: PLoS One. 2013 Jun 4;8(6):e65333. doi: 10.1371/journal.pone.0065333 (PMC3672088; doi:10.1371/journal.pone.0065333)

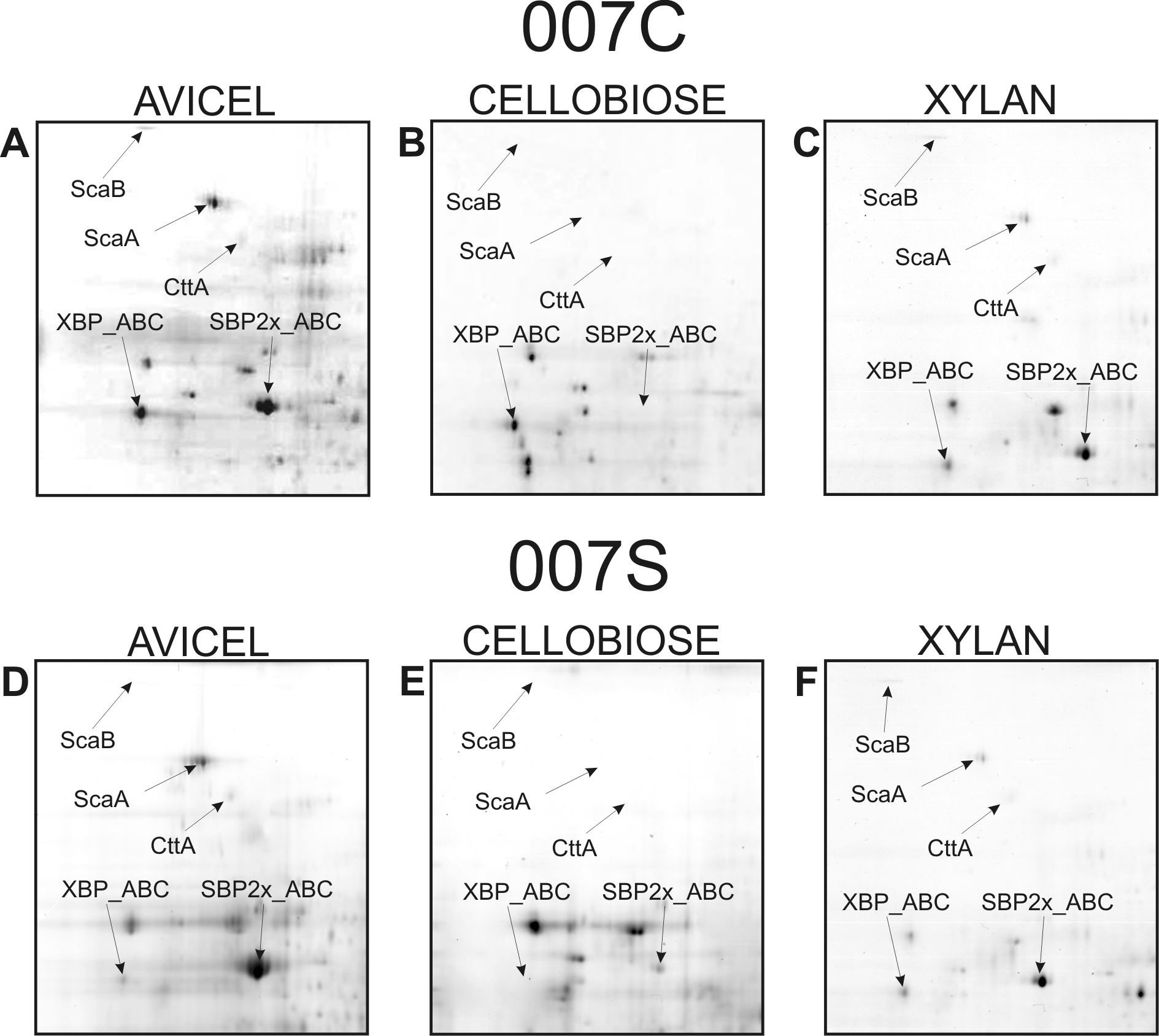

Supplement: Figure S2 — Major extracellular proteins identified in the cell wall associated (CWAP) fraction of R. flavefaciens 007C and 007S grown with cellobiose (24 h), xylan (48 h) or Avicel cellulose (7.5 d) as energy sources. Proteins referred to in Figure 4 are indicated by arrows. (TIF) [file pone.0065333.s002.tif]
