# Supplementary material for: Expression of Cellulosome Components and Type IV Pili within the Extracellular Proteome of Ruminococcus flavefaciens 007
Source: PLoS One. 2013 Jun 4;8(6):e65333. doi: 10.1371/journal.pone.0065333 (PMC3672088; doi:10.1371/journal.pone.0065333)

Figure S3. Clustal Ω alignment of N-terminal part of Pil3 (putative type IV pilin of *R. flavefaciens* 007C) with type IV pili from *Moraxella bovis*, *Pseudomonas* *aeruginosa*, *Dichelobacter nodosus*, *Neisseria meningitidis*, *Myxococcus xanthus*, *Eikenella corrodens*, *Neisseria gonorrhoeae* in *Aeromonas hydrophila.* Conserved amino acid (CAA) residues with nonpolar/hydrophobic residues are highlighted in blue, CAA with polar amino acid residues in green, CAA with negatively charged radicals in violet and positively charged in red. Conserved proline residues are highlighted in yellow and glycine in orange.


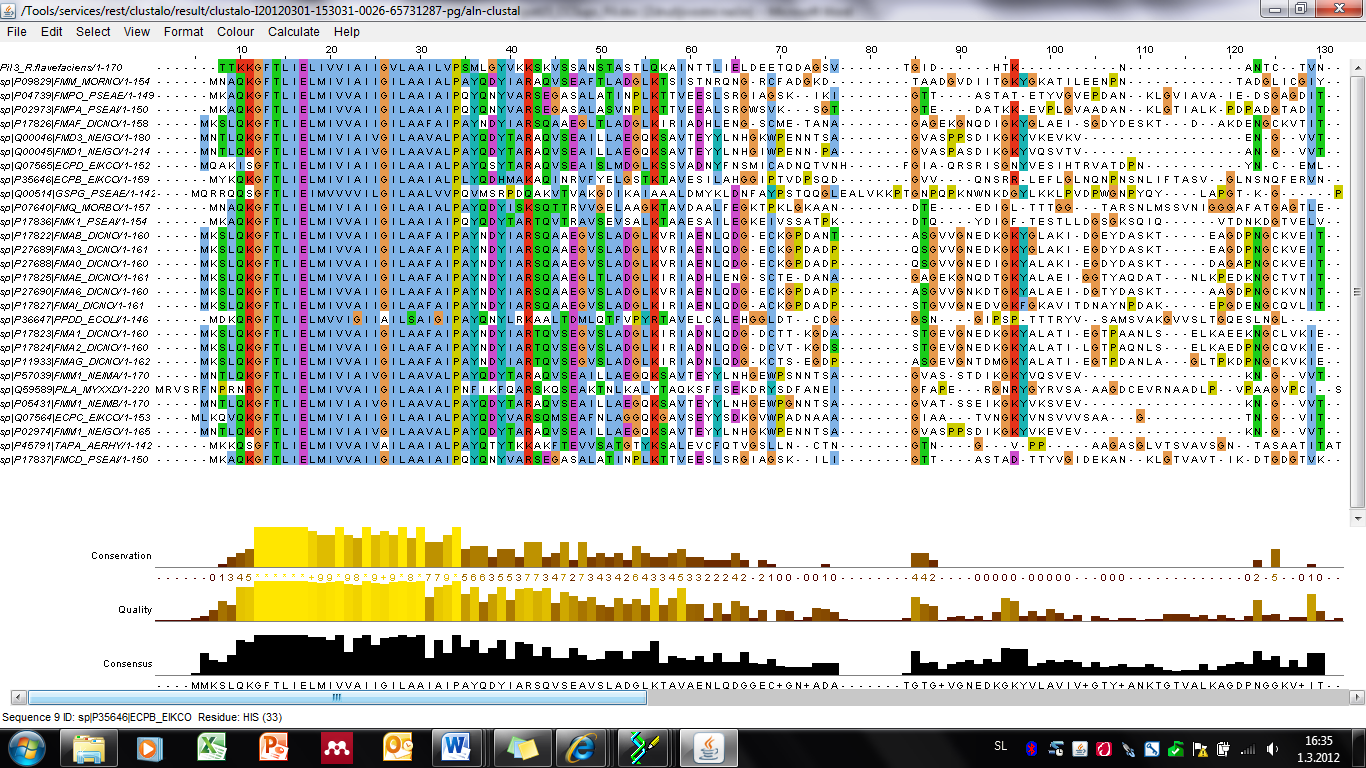

Supplement: Figure S3 — Clustal Ω alignment of N-terminal part of Pil3 (putative type IV pilin of R. flavefaciens 007C) with type IV pili from Moraxella bovis, Pseudomonas aeruginosa, Dichelobacter nodosus, Neisseria meningitidis, Myxococcus xanthus, Eikenella corrodens, Neisseria gonorrhoeae in Aeromonas hydrophila. (DOCX) [file pone.0065333.s003.docx]
