# Supplementary material for: Expression of Cellulosome Components and Type IV Pili within the Extracellular Proteome of Ruminococcus flavefaciens 007
Source: PLoS One. 2013 Jun 4;8(6):e65333. doi: 10.1371/journal.pone.0065333 (PMC3672088; doi:10.1371/journal.pone.0065333)
Supplement: Table S1 — Major proteins identified in the cell culture supernatant (CCSUP) fraction of R. flavefaciens 007C grown on Avicel for 7.5 days. (PDF) [file pone.0065333.s004.pdf]

Table S1. Major proteins identified in the cell culture supernatant (CCSUP) fraction of *R. flavefaciens* 007C grown on Avicel for 7.5 days. E-values apply to tBlastn scores acquired by matching »*de novo*« sequenced peptides to *R. flavefaciens* 007C open reading frames (ORFs). Theoretical masses and pIs are calculated for *R. flavefaciens* 007C proteins without signal sequences. See also main paper, Table 1.

Proteins identified by MASCOT search	Peptides matched	MASCOT score	Theoretical mass	Theoretical pI	Proteins with highest similarity	Identity (similarity)
ScaB scaffolding protein	18	1279	180956	4.28	CAC34385.1<br>CAO00730.1<br>ZP_06144574.1	98.9% (99.1%)<br>57.2% (82.4%)<br>43.4% (74.7%)
ScaA scaffolding protein	28	1239	89729	4.42	CAC34384.3<br>CAO00729.1<br>ZP_06144573.1	98.6% (99.2%)<br>45.8% (72.1%)<br>30.7% (58.3%)
Carbohydrate-binding protein CttA	31	1414	75007	4.53	CAH18995.2<br>CAO00731.1<br>ZP_06144575.1	98.3% (99.1%)<br>51.8% (80.2%)<br>44.2% (71.9%)
ScaC scaffolding protein	15	507	26168	4.51	CAE51046.2<br>CAQ16964.1<br>CAO00728.1<br>ZP_06144572.1	100.0% (100%)<br>75.5% (90.5%)<br>65.4% (87.9%)<br>52.0% (77.2%)
Glycoside hydrolase family 9 - Doc-1	4	202	108092	4.87	ZP_06141671.1(FD1)<br>TR:B9W4V4_RUMAL<br>TR:E9SHH5_RUMAL<br>TR:Q6TF33_RUMAL	73.0% (83.0%)<br>55.2% (74.9%)<br>55.3% (74.8%)<br>55.6% (74.7%)
Glycoside hydrolase family 48-Doc-1	5	165	91994	4.73	ZP_06145360.1(FD1)<br>TR:E9SAW3_RUMAL<br>TR:Q6TF32_RUMAL<br>TR:E6UFU2_RUMA7	67.0 % (78.0%)<br>48.7% (72.6%)<br>48.6% (72.6%)<br>47.6% (71.9%)
Rubrerythrin	5	240	20542	5.26	TR:D4JW52_9FIRM<br>TR:B0MQQ3_9FIRM<br>TR:D4MV01_9FIRM	82.4% (93.4%)<br>81.9% (92.9%)<br>77.0% (89.1%)
**Proteins identified by matching *"de novo"* sequenced peptides to *R. flavefaciens* 007C**	**Peptides matched**	**E-value**	**Theoretical mass**	**Theoretical pI**	**Proteins with highest similarity**	**Identity (similarity)**
Glycoside hydrolase family 26-Doc-1	4	1 e-004	80914	4.61	TR:E4MFR2_9FIRM<br>TR:E6UFF1_RUMA7<br>TR:D4JVP8_9FIRM	52.5% (77.4%)<br>41.2% (69.3%)<br>34.5% (68.4%)
Hypothetical protein with transmembrane regions	4	6 e-11	58160	4.38	TR:D6BDL2_9FUSO<br>TR:A5TTE3_FUSNP<br>TR:D4V0J6_ENTFA	24.3% (53.4%)<br>24.3% (54.4%)<br>24.3% (54.4%)
UgpB-like component of ABC-type sugar transport system (SBP2x_ABC)	32	1501	48199	4.61	TR:Q9S305_RUMFL<br>TR:D3AL57_9CLOT<br>TR:F4GLH9_9SPIO	100.0% (100%)<br>69.1% (87.1%)<br>68.0% (95.9%)
Xylose binding component of ABC-type sugar transporter system (XBP1_ABC)	2	0.02	43548	4.26	TR:F4XFN0_9FIRM<br>TR:A5ZMP8_9FIRM<br>TR:D4LQP2_9FIRM	64.9%(85.9%)<br>53.4%(83.8%)<br>63.1% (83.8%)
Protein with prepilin type IV N-terminal	3	3 e-006	13435	5.75	TR:D4JWE4_9FIRM<br>TR:Q573H1_RUMAL<br>TR:Q9Z4M8_RUMAL<br>TR:Q8KKF6_RUMAL	67.0% (91.4%)<br>55.6% (88.9%)<br>51.0 % (87.0%)<br>43.1 % (79.8%)
