# Supplementary material for: Expression of Cellulosome Components and Type IV Pili within the Extracellular Proteome of Ruminococcus flavefaciens 007
Source: PLoS One. 2013 Jun 4;8(6):e65333. doi: 10.1371/journal.pone.0065333 (PMC3672088; doi:10.1371/journal.pone.0065333)
Supplement: Table S2 — Major extracellular proteins identified in the cell wall associated (CWAP) fraction of R. flavefaciens 007C grown on Avicel for 7.5 days. (PDF) [file pone.0065333.s005.pdf]

Table S2. Major extracellular proteins identified in the cell wall associated (CWAP) fraction of *R. flavefaciens* 007C grown on Avicel for 7.5 days. E-values apply to tBlastn scores acquired by matching »*de novo*« sequenced peptides to *R. flavefaciens* 007C open reading frames (best matches). Theoretical masses and pIs are calculated for *R. flavefaciens* 007C proteins without signal sequences.

Proteins identified by MASCOT search	Peptides matched	MASCOT score	Theoretical mass	Theoretical pI	Proteins with highest similarity	Identity (similarity)
ScaA scaffolding protein	16	742	89729	4.42	CAC34384.3<br>CAO00729.1<br>ZP_06144573.1	98.6% (99.2%)<br>45.8% (72.1%)<br>30.7% (58.3%)
Carbohydrate-binding protein CttA	3	162	75007	4.53	CAH18995.2<br>CAO00731.1<br>ZP_06144575.1	98.3% (99.1%)<br>51.8% (80.2%)<br>44.2% (71.9%)
ScaC scaffolding protein	2	175	26168	4.51	CAE51046.2<br>CAQ16964.1<br>CAO00728.1<br>ZP_06144572.1	100% (100%)<br>75.5% (90.5%)<br>65.4% (87.9%)<br>52.0% (77.2%)
UgpB-like component of ABC-type sugar transport system (SBP2x_ABC)	32	1501	48199	4.61	TR:Q9S305_RUMFL<br>TR:D3AL57_9CLOT<br>TR:F4GLH9_9SPIO	100% (100%)<br>69.1% (87.1%)<br>68.0% (95.9%)
UgpB-like component of ABC-type sugar transport system (SBP1_ABC)	32	1501	60884	4.27	TR:D4LCF9_9FIRM<br>TR:D4LUD3_9FIRM<br>TR:E4MK92_9FIRM	44.3% (72.3%)<br>33.8% (68.8%)<br>36.0% (68.6%)
GroEL chaperonin	15	567	57230	4.98	TR:Q2KT44_RUMFL<br>TR:Q2KT43_RUMFL<br>TR:Q2KT40_RUMFL	98.0% (99.3%)<br>97.8% (99.1%)<br>96.7% (99.1%)
Immunogenic protein antigen 84	3	76	55206	5.24	TR:D4LDA9_9FIRM<br>TR:E3S7U2_PYRTT<br>SP:Y7557_DICDI	36.3% (62.5%)<br>24.9% (62.0%)<br>27.2% (61.8%)
Argininosuccinate sinthase	6	252	45723	5.11	TR:D4LFD7_9FIRM<br>TR:E6UB13_RUMA7<br>TR:E9SFS0_RUMAL	91.2% (97.1%)<br>86.7% (96.1%)<br>87.2% (97.1%)
Zn-dependent peptidase (M_16)	7	261	69070	4.79	TR:E7MKI7_9FIRM<br>TR:Q97EV0_CLOAB<br>TR:D4M699_9FIRM	46.4% (77.2%)<br>39.7% (71.2%)<br>37.6% (31.2%)
Enolase	5	267	46113	5.04	TR:E9S8R8_RUMAL<br>TR:E6UIF8_RUMA7<br>TR:E4MN84_9FIRM	93.8% (97.7%)<br>92.8% (97.0%)<br>89.8% (96.3%)
Phosphate acetyltransferase	3	114	35966	5.16	TR:E9SGG2_RUMAL<br>TR:E6UDK8_RUMA7<br>TR:E2ZIM6_9FIRM	92.6% (97.6%)<br>91.7% (96.7%)<br>70.2% (86.6%)
Fructose-bisphosphate aldolase	3	161	30229	5.08	TR:E6UGG0_RUMA7<br>TR:E9S948_RUMAL<br>TR:E4MIL1_9FIRM	94.4% (99.0%)<br>89.0% (96.6%)<br>84.3% (94.1%)
NifU homolog involved in Fe-S cluster formation	4	230	24734	5.00	TR:E9SDQ4_RUMAL<br>TR:C9L8K4_RUMHA<br>TR:F3AAF4_9FIRM	95.2% (98.7%)<br>92.2% (98.3%)<br>92.2% (98.3%)
Transcription elongation factor GreA	2	109	17384	4.8	TR:D4LD01_9FIRM<br>TR:E9S9L0_RUMAL<br>TR:E6UCK6_RUMA7	72.4% (86.5%)<br>60.1% (82.4%)<br>59.5% (82.4%)

Cell division initiation protein	3	129	20922	4.44	TR:D4LBS3_9FIRM<br>TR:B0PDZ2_9FIRM<br>TR:C0EEQ0_9CLOT	64.3% (83.2%)<br>35.8% (74.8%)<br>37.2% (73.8%)
Putative conserved protein with unknown function (DUF552)	1	59	21164	4.58	TR:D4LBR9_9FIRM<br>TR:D4L821_9FIRM<br>TR:E9SEM7_RUMAL	45.3% (75.1%)<br>35.1% (65.1%)<br>34.1% (65.1%)
Transcriptional regulator AraC	3	141	29241	5.81	TR:C4ICE8_CLOBU<br>TR:B1QUN4_CLOBU<br>TR:D3AUD9_9CLOT	29.3% (65.9%)<br>29.3% (65.9%)<br>31.5% (62.9%)
50S ribosomal protein L12P	16	402	12735	4.57	TR:D4LB62_9FIRM<br>TR:E9SF42_RUMAL<br>TR:E6UBP4_RUMA7	72.6% (95.2%)<br>74.4% (90.4%)<br>73.6% (90.4%)

**Proteins identified by *matching "de novo" sequenced peptides to R. flavefaciens* 007C**	**Peptides matched**	**E-value**	**Theoretical mass**	**Theoretical pI**	**Proteins with highest similarity**	**Identity (similarity)**
Xylose binding component of ABC-type sugar transport system (XBP1_ABC)	3	1 e-004	43548	4.26	TR:F4XFN0_9FIRM<br>TR:A5ZMP8_9FIRM<br>TR:D4LQP2_9FIRM	64.9% (85.9%)<br>53.4% (83.8%)<br>63.1% (83.8%)
Substrate binding component of ABC-type sugar transport system (SBP2_ABC)	2	6 e-0.38	58162	4.50	TR:C7GFK0_9FIRM<br>TR:D4LCF9_9FIRM<br>TR:D6DYI1_9FIRM	28.2% (65.9%)<br>33.3% (65.4%)<br>25.5% (63.7%)
