# Supplementary material for: Expression of Cellulosome Components and Type IV Pili within the Extracellular Proteome of Ruminococcus flavefaciens 007
Source: PLoS One. 2013 Jun 4;8(6):e65333. doi: 10.1371/journal.pone.0065333 (PMC3672088; doi:10.1371/journal.pone.0065333)
Supplement: Table S3 — Major extracellular proteins identified in cellulose-bound (CBP) fraction of R. flavefaciens 007C grown on Avicel for 7.5 days. (PDF) [file pone.0065333.s006.pdf]

Table S3. Major extracellular proteins identified in cellulose-bound (CBP) fraction of *R. flavefaciens* 007C grown on Avicel for 7.5 days. E-values apply to tBlastn scores acquired by matching »*de novo*« sequenced peptides to *R. flavefaciens* 007C open reading frames (best matches). Theoretical masses and pIs are calculated for *R. flavefaciens* 007C proteins without signal sequences.

Proteins identified by MASCOT search	Peptides matched	MASCOT score	Theoretical mass	Theoretical pI	Proteins with highest similarity	Identity (similarity)
ScaA scaffolding protein	16	742	89729	4.42	CAC34384.3<br>CAO00729.1<br>ZP_06144573.1	98.6% (99.2%)<br>45.8% (72.1%)<br>30.7% (58.3%)
Carbohydrate-binding protein CttA	42	1973	75007	4.53	CAH18995.2<br>CAO00731.1<br>ZP_06144575.1	98.3% (99.1%)<br>51.8% (80.2%)<br>44.2% (71.9%)
ScaC scaffolding protein	15	576	26168	4.51	CAE51046.2<br>CAQ16964.1<br>CAO00728.1<br>ZP_06144572.1	100% (100%)<br>75.5% (90.5%)<br>65.4% (87.9%)<br>52.0% (77.2%)
Glycoside hydrolase family 48-Doc1	3	202	91994	4.73	ZP_06145360<br>TR:E9SAW3_RUMAL<br>TR:Q6TF32_RUMAL<br>TR:E6UFU2_RUMA7	67.0% (78.0%)<br>48.7% (72.6%)<br>48.6% (72.6%)<br>47.6% (71.9%)
GroEL chaperonin	11	817	57230	4.98	TR:Q2KT44_RUMFL<br>TR:Q2KT43_RUMFL<br>TR:Q2KT40_RUMFL	98.0% (99.3%)<br>97.8% (99.1%)<br>96.7% (99.1%)
Phosphoglicerate kinase	7	392	42747	5.24	TR:E6UC03_RUMA7<br>TR:E9SBQ5_RUMAL<br>TR:D4MJJ1_9FIRM	85.7% (94.8%)<br>86.5% (94.3%)<br>85.7% (94.1%)
**Proteins identified by *matching "de novo" sequenced peptides to R. flavefaciens 007C***	**Peptides matched**	**E-value**	**Theoretical mass**	**Theoretical pI**	**Highest similarity score hits**	**Identity (similarity)**
Protein with Doc-1	2	3 e-37	32236	4.26	TR:C0EHK6_9CLOT<br>TR:A5ZAA6_9FIRM<br>TR:E1KG94_9FIRM	37.0% (65.4%)<br>43.7% (64.8%)<br>28.8% (62.2%)
ATP-dependent metalloprotease (FtsH)	4	2 e-0.12	51077	5.37	TR:E9SBX2_RUMAL<br>TR:E6UI87_RUMA7<br>TR:C0E9D5_9CLOT	69.8% (89.7%)<br>67.9% (88.0%)<br>68.2% (88.9%)
Hypothetical extracellular protein	3	7 e-004	22684	4.84	TR:C6RGL4_9PROT<br>TR:D0X4U2_VIBHA<br>TR:D0GLY8_9FUSO	28.7% (62.5%)<br>30.0% (60.8%)<br>28.9% (57.7%)
