# Supplementary material for: Expression of Cellulosome Components and Type IV Pili within the Extracellular Proteome of Ruminococcus flavefaciens 007
Source: PLoS One. 2013 Jun 4;8(6):e65333. doi: 10.1371/journal.pone.0065333 (PMC3672088; doi:10.1371/journal.pone.0065333)
Supplement: Table S4 — Major extracellular proteins identified in cellulose–bound (CBP) fraction of R. flavefaciens 007C grown on dewaxed cotton for 9.5 days. (PDF) [file pone.0065333.s007.pdf]

Table S4. Major extracellular proteins identified in cellulose–bound (CBP) fraction *R. flavefaciens* 007C grown on dewaxed cotton for 9.5 days. E-values apply to tBlastn scores acquired by matching »*de novo*« sequenced peptides to *R. flavefaciens* 007C open reading frames (best matches). Theoretical masses and pIs are calculated for *R. flavefaciens* 007C proteins without signal sequences.

**Proteins identified by MASCOT search**	**Peptides matched**	**MASCOT score**	**Theoretical mass**	**Theoretical pI**	**Highest similarity score hits**	**Identity (similarity)**
ScaA scaffolding protein	10	567	89729	4.42	CAC34384.3<br>CAO00729.1<br>ZP_06144573.1	98.6% (99.2%)<br>45.8% (72.1%)<br>30.7% (58.3%)
Carbohydrate-binding protein CttA	4	251	75007	4.53	CAH18995.2<br>CAO00731.1<br>ZP_06144575.1	98.3% (99.1%)<br>51.8% (80.2%)<br>44.2% (71.9%)
ScaC scaffolding protein	8	467	26168	4.51	CAE51046.2<br>CAQ16964.1<br>CAO00728.1<br>ZP_06144572.1	100% (100%)<br>75.5% (90.5%)<br>65.4% (87.9%)<br>52.0% (77.2%)
Glycoside hydrolase family 48-Doc1	3	256	91994	4.73	TR:E9SAW3_RUMAL<br>TR:Q6TF32_RUMAL<br>TR:E6UFU2_RUMA7	48.7% (72.6%)<br>48.6% (72.6%)<br>47.6% (71.9%)
Sporulation protein YtfJ	2	188	14813	4.56	TR:D4JUS2_9FIRM<br>TR:D4LCY6_9FIRM<br>TR:D4K3Y4_9FIRM	51.2% (86.2%)<br>57.5% (84.3%)<br>45.5% (84.3%)
**Proteins identified by *matching "de novo" sequenced peptides to R. flavefaciens 007C***	**Peptides matched:**	**E-value**	**Theoretical mass:**	**Theoretical pI:**	**Highest similarity score hits**	**Identity (similarity)**
Protein with Doc-1	2	5 e-17	32236	4.26	TR:C0EHK6_9CLOT<br>TR:A5ZAA6_9FIRM<br>TR:E1KG94_9FIRM	37.0% (65.4%)<br>43.7% (64.8%)<br>28.8% (62.2%)
Protein with prepilin type IV N-terminal	2	2 e-10	13435	5.75	TR:D4JWE4_9FIRM<br>TR:Q573H1_RUMAL<br>TR:Q9Z4M8_RUMAL<br>TR:Q8KKF6_RUMAL	67.0% (91.4%)<br>55.6% (88.9%)<br>51.0 % (87.0%)<br>43.1% (79.8%)
Thioredoxin	2	2 e-009	11767	4.39	TR:C9RMZ0_FIBSS<br>TR:F2JHL7_CELLD<br>TR:D4L5D4_9FIRM	63.1% (85.4%)<br>59.2% (81.6%)<br>57.4% (82.2%)
