# Supplementary material for: Expression of Cellulosome Components and Type IV Pili within the Extracellular Proteome of Ruminococcus flavefaciens 007
Source: PLoS One. 2013 Jun 4;8(6):e65333. doi: 10.1371/journal.pone.0065333 (PMC3672088; doi:10.1371/journal.pone.0065333)
Supplement: Table S5 — Major proteins identified in cell culture supernatant (CCSUP) fraction of R. flavefaciens 007C grown on dewaxed cotton for 9.5 days. (PDF) [file pone.0065333.s008.pdf]

Table S5. Major proteins identified in cell culture supernatant (CCSUP) fraction of *R. flavefaciens* 007C grown on dewaxed cotton for 9.5 days. E-values apply to tBlastn scores acquired by matching »*de novo*« sequenced peptides to *R. flavefaciens* 007C open reading frames (ORFs). Theoretical masses and pIs are calculated for *R. flavefaciens* 007C proteins without signal sequences.

Proteins identified by MASCOT search	Peptides matched	MASCOT score	Theoretical mass	Theoretical pI	Highest similarity hits	Identity (similarity)
ScaA scaffolding protein	19	986	89729	4.42	CAC34384.3<br>CAO00729.1<br>ZP_06144573.1	98.6% (99.2%)<br>45.8% (72.1%)<br>30.7% (58.3%)
Carbohydrate-binding protein CttA	15	821	75007	4.53	CAH18995.2<br>CAO00731.1<br>ZP_06144575.1	98.3% (99.1%)<br>51.8% (80.2%)<br>44.2% (71.9%)
ScaC scaffolding protein	5	385	26168	4.51	CAE51046.2<br>CAQ16964.1<br>CAO00728.1<br>ZP_06144572.1	100% (100%)<br>75.5% (90.5%)<br>65.4% (87.9%)<br>52.0% (77.2%)
Glycoside hydrolase family 48-Doc-1	2	193	91994	4.73	TR:E9SAW3_RUMAL<br>TR:Q6TF32_RUMAL<br>TR:E6UFU2_RUMA7	48.7% (72.6%)<br>48.6% (72.6%)<br>47.6% (71.9%)
Glycoside hydrolase family 9 - Doc-1	2	108	108092	4.87	TR:B9W4V4_RUMAL<br>TR:E9SHH5_RUMAL<br>TR:Q6TF33_RUMAL	55.2% (74.9%)<br>55.3% (74.8%)<br>55.6% (74.7%)
UgpB-like component of ABC-type sugar transport system (PBP2_LTTR superfamily)	32	1501	48199	4.61	TR:Q9S305_RUMFL<br>TR:D3AL57_9CLOT<br>TR:F4GLH9_9SPIO	100% (100%)<br>69.1% (87.1%)<br>68.0% (95.9%)
