# Supplementary material for: Expression of Cellulosome Components and Type IV Pili within the Extracellular Proteome of Ruminococcus flavefaciens 007
Source: PLoS One. 2013 Jun 4;8(6):e65333. doi: 10.1371/journal.pone.0065333 (PMC3672088; doi:10.1371/journal.pone.0065333)
Supplement: Table S6 — Type IV pili biogenesis cluster of R. flavefaciens 007C and three closest homologues of putative gene products. Abbreviations: SP - signal peptide, TMR(s) - transmembrane region(s). (PDF) [file pone.0065333.s009.pdf]

Table S6. Type IV pili biogenesis cluster of *R. flavefaciens* 007C and three closest homologues of putative gene products. Abbreviations: SP - signal peptide, TMR(s) - transmembrane region(s).

						**Blast**			**FASTA**	
**Contig**	**ORF**	**ORF coordinates**		**Annotation**	**Conserved domains**	**Homologues accession No.**	**E-value**	**Coverage**	**Highest similarty hits**	**Identity (similarity)**
00916	***pulF***	17502	18698	Type II secretion system protein	TMRs, Bacterial type II secretion system protein F domain (component PulF)	ZP_06142494.1<br>CBL16591.1<br>ZP_08158528.1	0.0<br>7e-158<br>3e-121	100%<br>99%<br>99%	TR:D4LAE6_9FIRM<br>TR:E9SAK1_RUMAL<br>TR:E6UE13_RUMA7	66.5% (88.2%)<br>56.4%(83.1%)<br>54.9%(83.1%)
00916	***pilM***	15878	17479	Type IV pilus assembly protein	TMR, Type IV pilus assembly protein, ATPase PilM Competence protein_A family.	ZP_06142493.1<br>CBL16592.1	0.0<br>2e-157	100%<br>96%	TR:D4LAE7_9FIRM<br>TR:E6UE12_RUMA7<br>TR:E9SAK0_RUMAL	56.0% (80.6%)<br>37.1% (73.0%)<br>36.6% (62.6%)
00916	***orfX***	12179	15010	Hypothetical protein	SP, TMR	ZP_06142491.1	4e-87	77%	TR:E5BYR1_9FUSO<br>TR:D4LAE9_9FIRM	30.3%(60.5%)<br>27.7%(56.6%)
00916	***pil1***	11672	12163	Protein with prepilin-type IV N-terminal domain	SP, TMR, pili subunits superfamily, prepilin-type N-terminal cleavage/methylation domain (type II secretory pathway, pseudopilin PulG)	ZP_06142490.1	2e-15	97%	TR:E6U2Z5_ETHHY<br>TR:A1SFQ5_NOCSJ<br>TR:D6KN13_9FIRM	37.3%(67.5%)<br>29.2% (66.0%)<br>33.8% (67.5%)
00916	***pil2***	10584	11657	Protein with prepilin-type IV N-terminal domain	SP, TMR, Prepilin-type N-terminal cleavage/methylation domain (type II secretory pathway, pseudopilin PulG / PulJ), EF-Hand 1 calcium-binding site	ZP_06142489.1<br>CBL16596.1<br>CBK97280.1	1e-25<br>3e-11<br>7e-04	97%<br>27%<br>16%	TR:D4JW11_9FIRM<br>TR:B0MQV6_9FIRM<br>TR:E6UE08_RUMA7	35.8% (79.0%)<br>33.7% (73.5%)<br>29.6% (73.2%)
00916	***pilD***	10514	9642	Prepilin peptidase (type IV leader peptidase)	SP, TMRs, peptidase A24A superfamily, prepilin type IV	ZP_06142488.1<br>CBL16597.1<br>YP_004105506.1	4e-121<br>3e-79<br>1e-58	98%<br>96%<br>93%	TR:D4LAF2_9FIRM<br>TR:E6UE07_RUMA7<br>TR:E9SAJ5_RUMAL	52.3% (80.5%)<br>45.1% (72.6%)<br>43.5% (73.9%)
00916	***pilB***	7822	9504	Pili biogenesis protein PilB-like ATPase, type II secretory pathway	Type II secretion system protein E, ATPase, AAA+ type core	ZP_06142487.1<br>CBL16598.1<br>ZP_08158500.1	0.00<br>0.00<br>0.00	100%<br>100%<br>100%	TR:D4LAF3_9FIRM<br>TR:E9SAJ4_RUMAL<br>TR:E6UE06_RUMA7	77.9% (92.2%)<br>63.0% (86.5%)<br>61.2% (85.4%)
00916	***pilT***	6677	7789	Pili biogenesis protein PilT-like ATPase, pilus retraction (twitching motility) protein	pilT_fam: twitching motility protein, P-loop containing nucleoside triphosphate hydrolases superfamily, ATPase, AAA+ type core	ZP_06142486.1<br>CBL16599.1<br>ZP_08158587.1	0.00<br>2e-164<br>2e-137	97%<br>96%<br>95%	TR:D4LAF4_9FIRM<br>TR:E9SAJ3_RUMAL<br>TR:E6UE05_RUMA7	79.2% (92.4%)<br>66.8% (87.6%)<br>66.8 % (87.6%)
00916	***pil3***	5665	6183	Protein with prepilin-type N-terminal cleavage/methylation domain	SP, TMR, pili subunits superfamily, prepilin-type N-terminal cleavage/methylation domain	ZP_06142477.1<br>CBL16600.1<br>CAC94922.1	1e-30<br>4e-21<br>6e-12	84%<br>81%<br>34%	TR:Q573H1_RUMAL<br>TR:Q9Z4M8_RUMAL<br>TR:Q8KKF6_RUMAL	55.6% (88.9%)<br>51.0 % (76.0 %)<br>43.1% (79.8%)
00916	***pil4***	4846	5493	Protein with prepilin-type IV N-terminal domain	SP, TMR, pili subunits superfamily, prepilin-type N-terminal cleavage/methylation domain	ZP_06142476.1<br>CBL16601.1<br>YP_004103555.1	9e-26<br>3e-14<br>3e-08	78 %<br>77 %<br>23 %	TR:E6UEG6_RUMA7<br>TR:Q9Z4M8<br>TR:E9SDT7_RUMAL	54.9% (90.2%)<br>52.8% (88.7%)<br>52.8% (88.7%)
